# New perspectives in neurosteroid action: open questions for future research

**DOI:** 10.3389/fncel.2014.00268

**Published:** 2014-09-19

**Authors:** Rainer Rupprecht

**Affiliations:** Department of Psychiatry and Psychotherapy, University of RegensburgRegensburg, Germany

**Keywords:** neurosteroid, allopregnanolone, TSPO, GABA, ion channel

Neurosteroids are still a hot topic in cellular and systemic neuroscience although the first report on anaesthetic actions of progesterone from Selye was published already in 1941 (Selye, 1941). It is a fascinating concept that endogenous metabolites of progesterone such as allopregnanolone and pregnanolone are powerful allosteric modulators of γ-aminobutyric acid type A (GABA_A_) receptors. This at a first glance simple principle created by nature raises several questions that still are major challenges for neurosteroid research. What is the exact site of interaction of such neurosteroids with GABA_A_ receptors? Is it really a binding site with clear saturable binding kinetics or rather an interaction site? Recent studies show that photolabeling of amino acids in the third transmembrane domain of the β 3 subunit of the GABA_A_ receptor by neurosteroid analogs is feasible (Chen et al., 2012) but does this really prove a binding site? What makes the difference in the regulation of GABAergic neurotransmission between the modulation by a 3α-reduced neurosteroid such as allopregnanolone and a benzodiazepine? Both are positive allosteric modulators of GABA_A_ receptors and enhance GABAergic neurotransmission but there appear to be great differences with regard to abuse liability and tolerance development (Rupprecht et al., 2009). Do they merely target different subunit compositions? An argument against this hypothesis is that allopregnanolone does not necessarily need a refined subunit composition to exert its actions, a β subunit is sufficient (Puia et al., 1990; Rupprecht and Holsboer, 1999). Thus, a more fascinating novel research area could be to identify what neuronal networks ultimately are targeted by either 3α-reduced neurosteroids or benzodiazepines. Do such neurosteroids and benzodiazepines recruit a different composition of post- and extrasynaptic GABA_A_ receptors? For example, future studies employing voltage sensitive dye imaging might address such questions. To what extent receptors other than GABA_A_ receptors are involved in neurosteroid action?

As a more systemical approach neuroimaging studies in humans, e.g., by means of functional magnetic resonance tomography (fMRI), might compare the brain areas involved after administration of benzodiazepines (Leicht et al., 2013) with neurosteroids such as allopregnanolone. As such, a major issue of future research in this area should be the elucidation of the mechanisms of action of neurosteroids both at the molecular, cellular and brain network level.

Another important area of research is the role of neurosteroids such as allopregnanolone for normal and pathological behavior in animals and humans and for neuropsychiatric disorders. It is evident from many preclinical studies that neurosteroids modulate anxiety-related behavior but nevertheless many issues are far from being understood. For example, what is the role of various neurosteroids with a different receptor profile acting in concert, e.g., pregnenolone and allopregnanolone? What about concentration and time dependency of neurosteroid effects? It may well be that such phenomena affect both physiological and pathological conditions. For example, it has been shown that negative mood symptoms may occur in women with premenstrual dysphoric disorder (PMDD) during the luteal phase of the menstrual cycle when progesterone and allopregnanolone levels usually are high (Bäckström et al., 2014) which has to be reconciled with the known anxiolytic effects of moderate to high concentrations of allopregnanolone. Moreover, in such patients there is an apparent discordance between the sensitivity to diazepam and allopregnanolone with decreased sensitivity to diazepam, whereas sensitivity to allopregnanolone is increased (Bäckström et al., 2014). A widely neglected research area is the role of isomers which acts as functional antagonists of allopreganolone, for example its 3β epimer (3β, 5α-pregnanolone). All these compounds finally act in concert in the modulation of rodent and human behavior. An example for such an altered equilibrium of steroid composition is the prominent decline in 3α-reduced neurosteroids after challenge with sodium lactate or cholecystokinin tetrapeptide (CCK-4) in patients with panic disorder together with a marked increase in the 3β-reduced isomer (Ströhle et al., 2003), which may result in a decreased GABAergic tone related to pathophysiology of panic attacks. Moreover, studies investigating the composition of neurosteroid profiles in neuropsychiatric disorders during differential psychopathological states are rare and need further elaboration. It is not surprising that neurosteroids such as allopregnanolone play a role in the pathophysiology of mood disorders (Schüle et al., 2014) and particularly for women (Schiller et al., 2014).

Besides their neuromodulatory potential a major issue is whether endogenous neurosteroids or synthetic neurosteroid derivatives can be used as novel therapeutic agents for the treatment of neuropsychiatric disorders. Ganaxolone is a first example of a synthetic 3α-reduced neurosteroid which is under investigation for the treatment of epilepsy, e.g., infantile spams (Riikonen, 2014). Another attractive area of research is the use of neurosteroidogenic compounds to promote endogenous neurosteroidogenesis. Observations came from both preclinical and clinical studies that for example antidepressants such as selective serotonin reuptake inhibitors (SSRIs) or mirtazapine (Pinna et al., 2006; Schüle et al., 2014) may enhance neurosteroidogenesis probably through interference with neurosteroidogenic enzymes. Moreover, ligands of the translocator protein 18 kDa (TSPO) have recently gained considerable attention as putative novel therapeutic agents in neuropsychopharmacology (Rupprecht et al., 2010). Numerous reports suggest that they promote the transport of cholesterol to the mitochondrial matrix thereby initiating neurosteroidogenesis, although recently the requirement of TSPO for steroidogenesis has been questioned (Morohaku et al., 2014; Tu et al., 2014). TSPO ligands are used as molecular imaging tools for assessing brain damage and microglia activation in positron emission tomography (PET) studies and have been suggested to exert potential beneficial effects in numerous preclinical investigations, for example peripheral nerve lesions (Rupprecht et al., 2010), neuropathic pain (Patte-Mensah et al., 2014), Alzheimer's disease (Chua et al., 2014), and retinal damage (Karlstetter et al., 2014). First clinical studies suggest that TSPO ligands, e.g., olesoxime, represent a therapeutic option in amyotrophic lateral sclerosis (Rupprecht et al., 2010). Moreover, TPSO ligands such as XBD173 or etifoxine may act as anxiolytic agents in clinical studies with a more favorable side effect profile than that of benzodiazepines (Rupprecht et al., 2009, 2010). It is intriguing that etifoxine is available in France since many years for the treatment of adjustment anxiety disorder. This shows that it is feasible to develop TSPO ligands for clinical indications with a favorable side effect profile.

In conclusion, neurosteroids, e.g., allopregnanolone, and neurosteroidogenic compounds such as TSPO ligands still represent a challenging area of research that has the potential to further elucidate the physiology of rodent and human behavior, the pathophysiology of neuropsychiatric diseases and to open the door for novel treatment avenues in neuropsychopharmacology.

## Conflict of interest statement

The author declares that the research was conducted in the absence of any commercial or financial relationships that could be construed as a potential conflict of interest.
